# Genomic differentiation across the speciation continuum in three hummingbird species pairs

**DOI:** 10.1186/s12862-020-01674-9

**Published:** 2020-09-03

**Authors:** Elisa C. Henderson, Alan Brelsford

**Affiliations:** Department of Evolution, Ecology, and Organismal Biology, University of California Riverside, 2710 Life Science Bldg, Riverside, CA 92521 USA

**Keywords:** Speciation genomics, Speciation continuum, Divergence, Polymorphism, Hummingbird, F_ST_, *d*_xy_

## Abstract

**Background:**

The study of speciation has expanded with the increasing availability and affordability of high-resolution genomic data. How the genome evolves throughout the process of divergence and which regions of the genome are responsible for causing and maintaining that divergence have been central questions in recent work. Here, we use three pairs of species from the recently diverged bee hummingbird clade to investigate differences in the genome at different stages of speciation, using divergence times as a proxy for the speciation continuum.

**Results:**

Population measures of relative differentiation between hybridizing species reveal that different chromosome types diverge at different stages of speciation. Using F_ST_ as our relative measure of differentiation we found that the sex chromosome shows signs of divergence early in speciation. Next, small autosomes (microchromosomes) accumulate highly diverged genomic regions, while the large autosomes (macrochromosomes) accumulate genomic regions of divergence at a later stage of speciation.

**Conclusions:**

Our finding that genomic windows of elevated F_ST_ accumulate on small autosomes earlier in speciation than on larger autosomes is counter to the prediction that F_ST_ increases with size of chromosome (i.e. with decreased recombination rate), and is not represented when weighted average F_ST_ per chromosome is compared with chromosome size. The results of this study suggest that multiple chromosome characteristics such as recombination rate and gene density combine to influence the genomic locations of signatures of divergence.

## Background

A fundamental goal in evolutionary biology is to understand how the process of speciation occurs. The increasing availability of population genomic data has led to a new understanding of speciation beyond the classical categorization of sympatric, allopatric, and parapatric modes [1]. The recently developed field of “speciation genomics” has revealed that speciation with gene flow, a phenomenon that was once thought to be highly unlikely [2], is common [3–7], including between extant and extinct taxa (reviewed in [8]). These revelations suggest that the individual is not the unit of isolation, and that there must be regions of isolation within the genome maintaining species boundaries. These sites are called barrier loci: specific regions of the genome that contribute to barriers to gene flow between populations [9]. This research has revealed that there are many genomic regions important to speciation, not just one or a few regions of large effect. For example, Martin et al. [10] found evidence of reduced introgression between two species of *Heliconius* butterflies at many regions of the genome, suggesting that many barrier loci of relatively small effect are responsible for maintaining species boundaries. Ellison et al. [11] found evidence that many genes of small effect are responsible for the divergence of sexual behaviors between species of *Laupala* crickets. Overall, investigating the genomic landscapes of differentiation between hybridizing species using modern genomics techniques will enhance our understanding of speciation [1, 12].

The study of genomic divergence was initially focused on the population statistic, F_ST_, which is the standard statistical measure for genetic divergence between two populations and is based on between-population variance in allele frequencies compared to within-population variance [13]. It was observed early on that F_ST_ is variable among loci [14]. Studies have since shown that the variation in F_ST_ creates a heterogeneous landscape across the genome. This pattern has been observed in several diverse taxon pairs, including *Heliconius* butterflies [15], mussels [16], warblers [17, 18], and *Ficedula* flycatchers [19]. Originally, it was thought that peaks of differentiation were indicative of reproductively isolating genes [20], and were termed “genomic islands of speciation” [21]. Further scrutiny revealed that equating F_ST_ peaks to “islands of speciation” (i.e. barrier loci) is premature, and additional investigation is required to determine the true cause of F_ST_ heterogeneity [9, 22–24].

Though genome-wide average F_ST_ increases as divergence time increases [25, 26] it is clear that F_ST_ peaks relative to the rest of the genome are not necessarily indicative of barrier loci or reduced gene flow, and a variety of processes may lead to these “outlier regions”. Recombination rate variation across the genome predicts much of the variation in nucleotide diversity [27] and F_ST_ [28–30]. Reduced recombination rate, genetic drift, local adaptation, and other evolutionary processes may reduce local nucleotide diversity within at least one species in the pair causing peaks in F_ST_ that are not indicative of locally reduced gene flow [22, 23]. In contrast to F_ST_, an absolute measure of divergence between populations (*d*_xy_) is not inflated by reduced within-population nucleotide diversity. F_ST_ peaks resulting from locally reduced gene flow are predicted to have elevated *d*_xy_, while F_ST_ peaks resulting from low within-population diversity are not. Thus, comparing multiple statistics together may help elucidate the evolutionary mechanisms leading to the genomic patterns we find. For example, measuring nucleotide diversity (e.g. π) across the genome can indicate specific regions of low diversity in one or both species that result in an F_ST_ peak that is not due to reduced gene flow, but rather due to species-specific selection at that locus. Calculating *d*_xy_ across the genome and locating F_ST_ peaks that are associated with elevated *d*_xy_ can help narrow down the potential causes of some islands of divergence (e.g. [18, 31–34]).

Though much progress has been made in characterizing the heterogeneity in divergence using these statistics, our understanding of how these patterns change over time is still limited. Because speciation is often a process with a duration of at least 1 million years [35], it is nearly impossible to investigate the different stages of speciation using only a single species pair. To help alleviate this problem, some studies have used independent pairs of closely related species that have different divergence times as a proxy for the different stages of speciation (e.g. [15, 18, 26, 28, 31, 36–40]). This can provide valuable insight into the genomic process of speciation over time.

Variation in chromosome type may be important to consider when investigating the speciation process. For example, sex chromosomes play a disproportionate role in reproductive isolation relative to the autosomes [41]. Greater differentiation on sex chromosomes relative to autosomes has been broadly identified in both male-heterogametic (XY/XX) and female-heterogametic (ZW/ZZ) taxa [42]. Proposed reasons for this include the large X-effect, reduced effective population size, and reduced recombination rate of X or Z chromosomes [42]. Chromosome size variation across autosomes may also contribute to the process of speciation. All birds and many species of reptiles have a largely conserved karyotype made up of large chromosomes (macrochromosomes) and small chromosomes (microchromosomes) [43]. Often, the karyotype contains up to 8 large chromosomes that are on average an order of magnitude larger than the average microchromosome [44]. Microchromosomes have a higher recombination rate, gene density, and GC content relative to macrochromosomes [43]. Low recombination rates are predicted to lead to reduced genetic diversity due to hitchhiking [45] and background selection [46, 47]. Consistent with this prediction, in birds larger chromosomes tend to have lower diversity [48, 49] and, in at least one case, higher F_ST_ between lineages [50]. Whether microchromosomes play a different role in speciation than macrochromosomes, however, is still unknown.

**Fig. 1:**
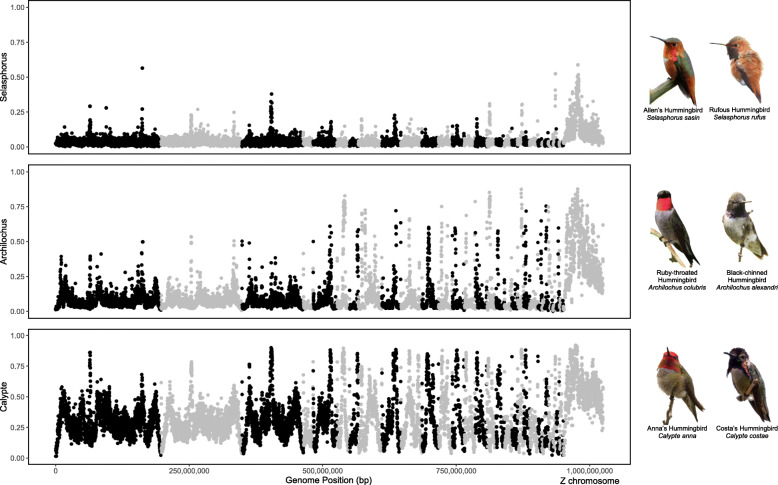
F_ST_ calculated in 100 kbp windows across the whole genome for three species pairs. Three pairs of hybridizing species have different divergence times (estimated by [51]): top (*Selasphorus*; 0.97 my), middle (*Achilochus*; 1.5 my), bottom (*Calypte*; 2.52 my). Chromosomes alternate in color. Z chromosome (right) has increased F_ST_ relative to autosomes for all three species pairs. Photograph credits: *S. sasin* by M. Shattock, CC-BY-SA 2.0; *S. rufus* by Kaaren Perry, CC-BY 2.0; *A. colubris* by Dick Ledbetter; *A. alexandri* by Bill Shreve; *C. anna* by Becky Matsubara, CC-BY 2.0; *C. costae* by Daniel Pierce

In this study, we investigate 1) how genomic signatures of divergence change as speciation proceeds, and 2) the differences between micro-, macro-, and Z chromosomes, how those differences compare across the speciation continuum, and what that tells us about the importance of different chromosome types in speciation. We use three pairs of hybridizing species from the bee hummingbird clade: *Calypte anna* and *C. costae* (Anna’s and Costa’s hummingbirds), *Archilochus alexandri* and *A. colubris* (Black-chinned and Ruby-throated hummingbirds), *Selasphorus sasin* and *S. rufus* (Allen’s and Rufous hummingbirds; Fig. 1). Previous studies have used multiple pairs of species with different divergence times as a proxy for the speciation continuum (e.g. [15, 18, 26, 28, 31, 36–40]). However, recent studies on the phylogenetic relationships among these hummingbirds have produced conflicting estimates of relative divergence times. According to McGuire et al. [51] the species pairs *S. sasin*/*S. rufus* and *A. alexandri*/*A. colubris* diverged more recently than *C. anna*/*C. costae*, but numerical estimates of divergence dates are not provided. Abrahamczyk and Renner [52] is the only study to our knowledge to provide numerical estimates for the three species pairs included in this study: *C. anna*/*C. costae* estimated at 2.52 million years ago (mya), *A. alexandri*/*A. colubris* 1.5 mya, and *S. sasin/S.rufus* 0.97 mya. Licona-Vera and Ornelas [53] used improved within-species sampling (previous studies included only a single representative of four [51] or six [52] of our focal species), and did not recover monophyletic groups for *A. alexandri*, *S. sasin*, or *S. rufus*. The latter study also estimated an older divergence date for a node within *A. alexandri* than the node separating *C. anna* and *C. costae*, albeit with overlapping 95% HPD intervals. Because of the lack of consensus in the literature on divergence times and phylogenetic relationships among the three focal species pairs of this study, we chose to use extent of reproductive isolation (i.e., frequency of hybridization), rather than divergence time, as a proxy for the speciation continuum.

Our species pairs are especially suitable for this study, as *A. alexandri*/*A. colubris* and *S. sasin*/*S. rufus* are, to our knowledge, the only two hummingbird species pairs in the US with a quantified extent of hybridization. *A. alexandri* and *A. colubris* have a narrow sympatric range in southwestern Oklahoma, and an estimated 9.3% of adult males are F1 hybrids [54]. *S. sasin* and *S. rufus* have a broad hybrid zone in northern California and southern Oregon where hybrids outnumber parental species and there is clinal variation in species diagnostic traits [55]. *C. anna* and *C. costae* inhabit a broad sympatric range in parts of California, Nevada, Arizona, and Baja California. Though the frequency of hybridization has not been quantified, hybrids are occasionally observed [[56], pers. obs.], and this is the only species pair in the United States listed as “extensive natural hybridization reported” by [57]. Species pairs will hereafter be referred to by genus (*Calypte, Archilochus, Selasphorus*).

We use these three independent but closely related species pairs as a proxy for the speciation continuum. First, we compare patterns of genomic differentiation and diversity across these three levels of reproductive isolation. Second, we compare these patterns across three different chromosome types: microchromosomes, macrochromosomes, and the sex chromosome, and observe how these comparisons differ across the three different levels of reproductive isolation. We found that speciation seems to progress at different rates based on chromosome type, with the sex chromosome diverging first, the microchromosomes diverging next, and divergence only appearing on the macrochromosomes in late stages of reproductive isolation.

## Results

**Fig. 2:**
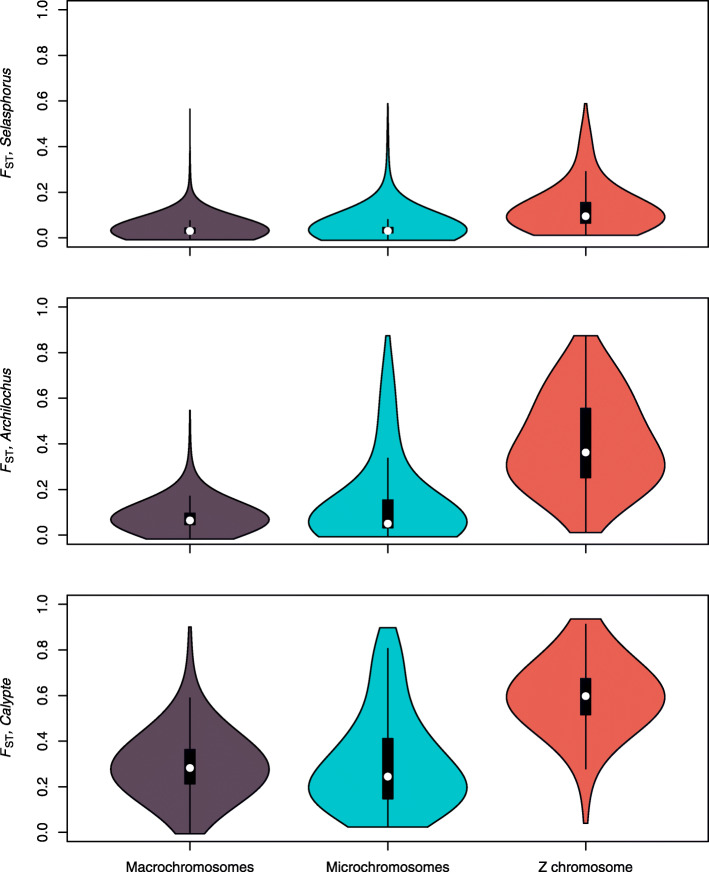
Density of windows with different F_ST_ values separated by chromosome type

Our least reproductively-isolated species pair, *Selasphorus* had the lowest average F_ST_ (0.041) and fewer overall F_ST_ peaks than either of the other species pairs (Fig. 1). Our most reproductively-isolated species pair, *Calypte* had the highest average F_ST_ (*Calypte,* F_ST_ = 0.323; *Archilochus,* F_ST_ = 0.112), though *Calypte* and *Archilochus* had a qualitatively similar number of F_ST_ peaks. For all three pairs, F_ST_ was higher on the Z chromosome than on the autosomes, with a significant overrepresentation of high-F_ST_ windows on the Z chromosome (*Calypte,* χ^2^ = 40.975, *P* = 1.27 × 10^− 9^; *Archilochus,* χ^2^ = 292.91, *P* = 2.2 × 10^− 16^; *Selasphorus,* χ^2^ = 674.01, *P* = 2.2 × 10^− 16^; Supplemental Table 1), consistent with the findings of Battey [58] in the *Selasphorus* species pair and Elgvin et al. in *Passer* sparrows [59]. When comparing F_ST_ across chromosome types, *Selasphorus,* the least-reproductively isolated species pair only had noticeably elevated F_ST_ on the Z chromosome, and not on autosomes, and no significant difference in number of elevated F_ST_ peaks on microchromosomes versus macrochromosomes (χ^2^ = 0.09577, *P* = 0.757). The next species pair, *Archilochus* showed elevated F_ST_ on the Z chromosome and significantly more elevated F_ST_ windows on the microchromosomes relative to the macrochromosomes (χ^2^ = 48.998, *P* = 2.56 × 10^− 12^). The most reproductively-isolated species pair, *Calypte* had windows with elevated F_ST_ throughout the entire genome, including the macrochromosomes (Fig. 2), though the microchromosomes had significantly more elevated F_ST_ windows than the macrochromosomes (χ^2^ = 22.759, *P* = 1.27 × 10^− 9^). Genes found within the top 1% of F_ST_ peaks are listed in Supplemental Table 2, although we emphasize that not all high-F_ST_ regions should be interpreted as barrier loci, and this table likely includes many genes that have no involvement in reproductive barriers.

**Fig. 3:**
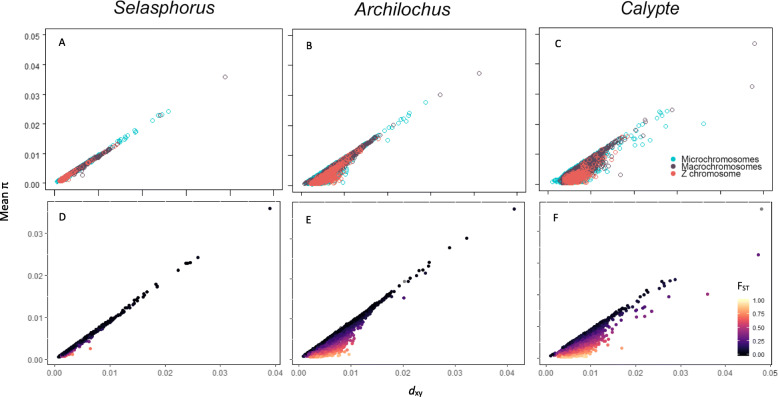
Absolute divergence (*d*_xy_) versus mean nucleotide diversity (π) calculated in 100 kbp windows. *d*_xy_ and π were positively correlated in each species pair and chromosome type (**a**-**c**). Windows with high F_ST_ tend to fall in regions with low π relative to *d*_xy_ (**d**-**e**)

**Fig. 4:**
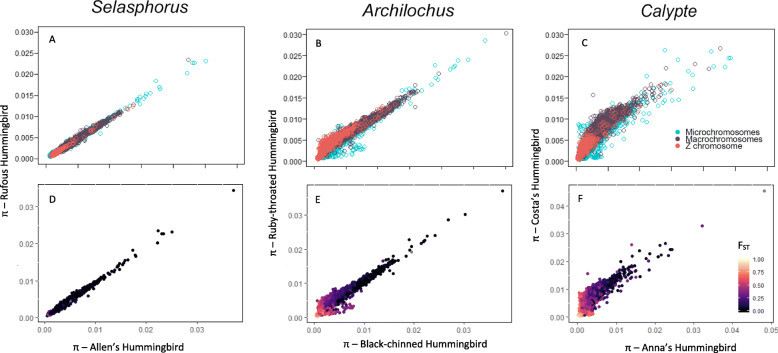
π versus π between hybridizing species calculated in 100 kbp windows. π was strongly positively correlated with π of its hybridizing species across all chromosome types (**a**-**c**). Windows with high F_ST_ are located where π for both species is low (light blue = high F_ST_, dark blue = low F_ST_; **d**-**e**)

For each species pair, F_ST_ and *d*_xy_ were negatively correlated (Supplemental Fig. 1). The pattern held true across all three chromosome types, but the strength of the positive correlation did not vary consistently (Supplemental Fig. 1). Within species pairs, there was always a positive correlation between *d*_xy_ and mean π for all three chromosome types (Fig. 3). The correlation was strongest for the least reproductively-isolated species pair (*Selasphorus*; Fig. 3a). The correlation was weaker for the next species pair (*Archilochus*), with increased *d*_xy_ relative to π appearing only on the Z chromosome (Fig. 3b). Increased *d*_xy_ relative to π appeared on all chromosome types in the most reproductively-isolated species pair (*Calypte*; Fig. 3c). Windows with elevated F_ST_ appeared in regions with higher *d*_xy_ relative to π, or where π was especially low (Fig. 3d-f). π was strongly positively correlated between hybridizing species (Fig. 4a-c). Elevated F_ST_ appeared mostly in windows where both species in a pair had low π (Fig. 4d-f).

**Fig. 5:**
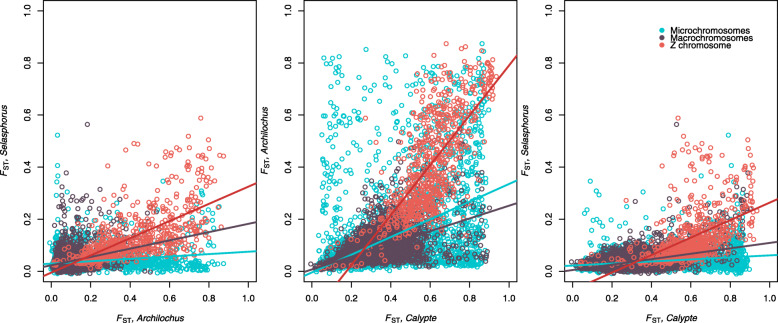
F_ST_ for one species pair versus F_ST_ for another species pair calculated in 100 kbp windows. F_ST_ is positively correlated across all species pairs and all chromosome types

**Fig. 6:**
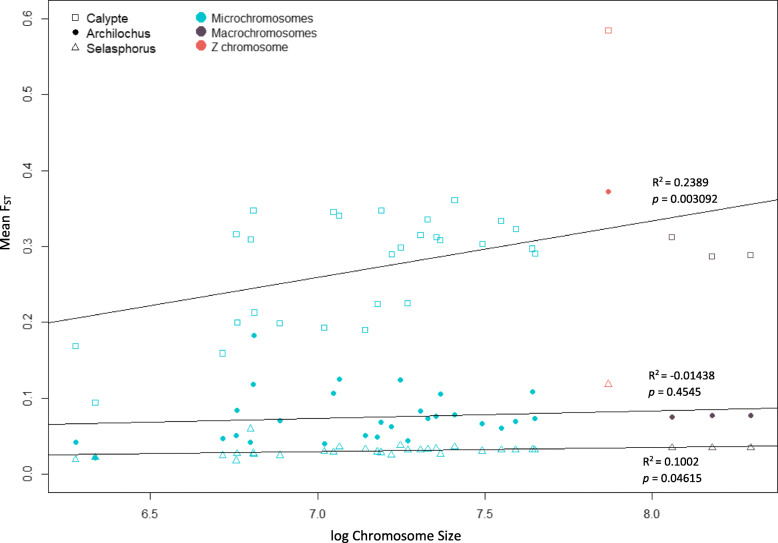
F_ST_ versus chromosome size. Mean F_ST_ increased with chromosome size only for the most divergent species pair (*Calypte*). Regression lines, *p*-values and *R*^*2*^ are calculated for all autosomes, excluding the Z chromosome

Across species pairs, F_ST_ from one pair was always positively correlated with F_ST_ from either of the other species pairs (Fig. 5a-c; Table 1), indicating that patterns of F_ST_ peaks and valleys in one species pair can partially predict patterns in another species pair. The pattern was true for all chromosome types, but the strength in correlation did not vary consistently (Table 1; Supplemental Fig. 2). F_ST_ increased with chromosome size for the most reproductively-isolated species pair (*Calypte*; Fig. 6), with a weaker positive correlation for the less reproductively-isolated species pairs (*Selasphorus* and *Archilochus*; Fig. 6).

**Table 1:** F_ST_ across species pairs is positively correlated for all chromosome types

Genus 1 (x)	Genus 2 (y)	Chrom type	R-squared
*Archilochus*	*Selasphorus*	Macro	0.0863347
		Micro	0.0522726
		Z	0.4069244
*Calypte*	*Archilochus*	Macro	0.3053203
		Micro	0.2404795
		Z	0.5296113
*Calypte*	*Selasphorus*	Macro	0.1883183
		Micro	0.0801977
		Z	0.2432459

## Discussion

In this study we compared the genomic differentiation and diversity of three pairs of closely related hummingbirds at different stages of divergence (*Selasphorus*, extensive hybridization [55]; *Archilochus,* moderate hybridization [54], *Calypte*, rare hybridization [56, 57];), allowing us to investigate changes in the genome as divergence progresses. Our estimates of F_ST_ are consistent with one previously published estimate of divergence times [52] for these three species pairs (Fig. 1), suggesting that both average levels of F_ST_ and number of F_ST_ peaks increase as extent of reproductive isolation and divergence time increase.

Chromosome-wide average F_ST_ increased with chromosome size for our most reproductively-isolated species pair (*Calypte*; Fig. 6) as is theoretically expected given the correlation between chromosome size and recombination rate in birds [22, 60] and empirically tested in at least one other species [50]. Finding significance of this pattern in *Calypte* and not in the other species pairs suggests that it may be a pattern that appears late in speciation. However, we cannot exclude that differences in natural history among the species pairs contributes to our results. For example, the *Calypte* species are the only hummingbirds in this study that are not long-distance migrants (sedentary populations of *S. sasin* exist, but were not sequenced). How natural history characteristics such as migration affect genomic patterns of speciation is unclear.

When looking at F_ST_ in windows across the genome, we did not find the expected pattern of increased F_ST_ with increased chromosome size. Windows with high F_ST_ accumulated on the microchromosomes earlier in speciation than on the macrochromosomes (Fig. 2). This pattern is unexpected given the reduced recombination rate on macrochromosomes relative to microchromosomes, and it was not predictable from the correlation between F_ST_ and chromosome size. F_ST_ is expected to increase in regions of the genome that have reduced rates of recombination because the inheritance of linked loci results in locally reduced diversity within species over time. Likewise, F_ST_ is expected to decrease where recombination rate is high, because nucleotide diversity will increase in these regions over time. These predictions bring to light the peculiarity of our result that F_ST_ peaks seemed to accumulate on microchromosomes, where recombination rate is high, earlier in the divergence process than macrochromosomes, where recombination rate is lower. Average F_ST_ did not increase on small chromosomes relative to large chromosomes, but rather 100 kbp windows with elevated F_ST_ were more common on microchromosomes than on macrochromosomes in early stages of speciation. The early accumulation of F_ST_ peaks on microchromosomes may be due to a combination of characteristics of these small chromosomes. For example, higher gene density on microchromosomes may provide more targets for positive selection to act on, and higher recombination rate may increase the efficiency of selection in fixing beneficial mutations by reducing Hill-Robertson interference. If the rate of adaptive evolution is higher on microchromosomes, this could lead to an earlier accumulation of F_ST_ peaks either as a direct result of within-species selective sweeps, or because some of these selective sweeps result in reproductive barrier loci. High linkage on macrochromosomes results in reduced diversity which leads to the accumulation of F_ST_ peaks on macrochromosomes observed at later stages of speciation.

Elevated F_ST_ windows were especially common on the Z chromosome in all three species, as expected based on prior work in hummingbirds [58] and other taxa [42]. The large X-effect, or the observation that sex chromosomes play a disproportionate role in speciation, is thought to be the overarching cause of the commonly found pattern of elevated F_ST_ on sex chromosomes relative to autosomes. Hypothesized reasons for the large X-effect include ploidy difference between sexes, faster evolution of the sex chromosomes and higher density of hybrid incompatibility loci on the sex chromosomes (reviewed in [42]). Using our data, we cannot distinguish between these processes nor determine whether the sexual dimorphism observed in hummingbirds plays a strong role in this genetic pattern.

Absolute divergence (*d*_xy_) and nucleotide diversity (π) were strongly positively correlated and relative divergence (F_ST_) was negatively correlated with *d*_xy_ in all three species pairs. This suggests a pattern of selection before divergence wherein pre-speciation selection causes regions of reduced *d*_xy_ and low diversity (π). Repeated linked selection at these regions before and after speciation can cause locally elevated relative divergence (F_ST_) despite reduced absolute divergence (*d*_xy_) [23]. Alternatively, this pattern could result from a global cross-species selective sweep after divergence (“sweep-before-differentiation;” [37]) at loci with elevated F_ST_ (e.g. [61]). This pattern is consistent with some previous findings in other species pairs of birds [62, 63]. However, given that the species in this study are the result of a recent rapid radiation and have recent common ancestors, a measure of absolute divergence might be unreliable for determining the evolutionary history of species pairs with such recent divergence times. *d*_xy_ measures the nucleotide differences that have accumulated since the divergence of the two focal species, but also reflects ancestral polymorphism that was present before divergence. Therefore, species pairs that have not been diverging for very long (including all three focal pairs of this study) are expected to have nucleotide diversity that is at least partially representative of ancestral polymorphism. Thus, *d*_xy_ that strongly correlates with π for these species pairs may indicate that much of the polymorphism in the ancestor of each species pair is retained in the extant populations [22].

The strong correlation between π and *d*_xy_ was present in all three species pairs, but the relationship weakened as extent of reproductive isolation increased, indicating that absolute divergence increases relative to levels of within-species diversity over the course of the speciation process. F_ST_ peaks appearing in windows that have higher *d*_xy_ relative to π is expected, given that F_ST_ is a measure of differentiation relative to within-species polymorphism (Fig. 3d-f).

Nucleotide diversity (π) was strongly correlated between hybridizing species (Fig. 4a-c) and the relationship was stronger for species pairs with less reproductive isolation. In species pairs with greater reproductive isolation the correlation weakened, with some genomic windows showing reduced π in one but not both species (Fig. 4b-c). Elevated F_ST_ in windows where one species has low nucleotide diversity relative to its closest relative is expected to be caused by within-species selection, rather than divergent selection between the species. However, we did not find strong evidence of elevated F_ST_ being caused by reduced polymorphism in one species within a pair as F_ST_ peaks appeared primarily on windows where both species had reduced π (Fig. 4d-f). A positive correlation of F_ST_ across species pairs might indicate that the landscape is partially driven by genomic features such as local recombination rate that are conserved across a higher phylogenetic level [63].

Differences in the genetic signatures of speciation across species pairs may be attributable to the natural history and phenotypic differences among species. For example, differences in plumage color are weak between Allen’s and Rufous hummingbird (genus *Selasphorus*), and these species are commonly misidentified. By contrast, the species pairs in *Calypte* and *Archilochus* have distinct differences in male plumage color, especially in the gorget feathers. Additionally, mating displays, habitats and migration habits differ across the six species in this study. Anna’s and Costa’s hummingbirds (genus *Calypte*) have complicated and poorly known migration patterns, with variation across populations and movement that is likely driven by variation in availability of food [64, 65]. Black-chinned (genus *Archilochus*), Ruby-throated (genus *Archilochus*) and Rufous hummingbirds perform complete migrations [66–68], while Allen’s hummingbirds include both migratory and sedentary populations [69]. Demographic differences across the species, such as historical changes in population size and distribution could also affect genetic signals that we interpret as signatures of speciation. For example, Anna’s hummingbird has drastically increased its breeding range in the last 100 years, likely as a result of increased availability of food in the form of exotic plants and hummingbird feeders [70]. In Allen’s hummingbird, the non-migratory subspecies (*S. s. sedentarius*) has expanded its breeding range into mainland southern California from the Channel Islands [71]. While the breeding ranges for other species used in this study seem to have remained unchanged over time, observations of birds wintering over an expanded range have been observed [63–65]. These differences across the species used in this study highlight that each species pair is subject to its own evolutionary trajectory leading to a unique speciation event. While this is a general caveat of using independent species pairs as a proxy for the speciation continuum, we believe that the differences we observe among chromosome types can inform the ongoing debate about the roles of selection and recombination in the genetics of speciation.

## Conclusions

In this study we found evidence for the earlier divergence of microchromosomes than macrochromosomes in speciation through comparing genomic differentiation and diversity across the speciation continuum. Our study is the first, to our knowledge, to compare genomic statistics across different categories of autosomes and across independent, closely related species pairs with different levels of reproductive isolation. The results of this study suggest that variation in chromosome size, or in associated characteristics such as recombination rate and gene density, plays an important role in determining the genomic landscape of divergence at different points along the speciation continuum.

## Methods

### Sampling, extractions and sequencing

We collected samples from populations of three species pairs of hummingbirds for a total of six species and 59 individuals (Supplemental Table 3): (1) Allen’s and Rufous (*Selasphorus sasin*, 9 samples; *S. rufus*, 7 samples), (2) Anna’s and Costa’s (*Calypte anna*, 12 samples; *C. costae*, 12 samples), and (3) Black-chinned and Ruby-throated (*Archilochus alexandri*, 10 samples; *A. colubris*, 9 samples). All populations were collected from allopatric regions with the exception of the *Calypte* species pair which was collected from the sympatric range in Riverside, CA. *C. anna* and *C. costae* hybridize infrequently, but the sympatric sampling for this species pair may lead to an underestimate of genetic differentiation between these species.

Samples from *Calypte* and *Selasphorus rufus* populations were extracted from dried blood spots on filter paper using the Qiagen DNeasy extraction protocol. Samples from both *Archilochus* species were acquired from Dr. Chris Clark’s collected samples stored at the Yale Peabody Museum of Natural History, and samples from *Selasphorus sasin* were provided by the California Academy of Sciences. Small pieces of tissue were then extracted using the DNeasy extraction protocol. All DNA concentrations were quantified using a Qubit fluorometer and then diluted to 4 ng/uL in preparation for a modified Nextera Whole Genome Library prep protocol [[72]; see Supplementary Material for our modifications to that protocol].

### Alignment and SNP calling

We used the Burrow-wheeler aligner (BWA-mem [73];) to align the sequences to an Anna’s Hummingbird reference genome [74, 75]. We called variants using Samtools mpileup (v1.8 [76];) and filtered nucleotide positions for missing data (20% per locus, −-max-missing 0.8), minimum depth (−-minDP 2), biallelic sites (−-maxalleles 2), and removed indels using VCFtools (v1.15 [77];), retaining all positions passing these filters, including invariant sites.

### Population statistics

We used VCFtools to calculate allele frequency and Weir and Cockerham’s [78] weighted F_ST_. Allele frequency was calculated for each nucleotide position passing our depth and missingness filters and F_ST_ was calculated for each non-overlapping 100 kbp window across the genome. From allele frequency, π and *d*_xy_ were calculated for each SNP following [37]. The result per SNP was then averaged over 100 kbp windows to match the windows in which F_ST_ was calculated. Separate files for π, *d*_xy_ and F_ST_ were combined such that any window with a missing value for any one of the statistics was excluded from the final file.

We classified chromosomes 1, 2 and 3 as macrochromosomes and chromosomes 4–33 as microchromosomes. Though size varies across all chromosomes, the first three average an order of magnitude larger than the rest of the autosomes, and combined account for nearly one half of the entire genome size. For comparisons across the different chromosome types after filtering and SNP-calling, we separated the genome into three parts: microchromosomes, macrochromosomes, and Z chromosome. For analyses on the Z chromosome we used only the male individuals for population statistics calculations. Following Elgvin et al. [59] we compared the distribution of high-F_ST_ regions (100 kbp windows in the top 1% for each species pair) among macrochromosomes, microchromosomes, and the Z chromosome using a chi-squared test in R v3.4.3. We used the Bedtools v2.28 [79] intersect command with the “-loj” option to obtain a list of annotated genes overlapping windows in the top 1% of F_ST_ values for each species pair.

## Supplementary information

**Additional file 1: Supplemental Fig. 1.** *d*_xy_ vs F_ST._

**Additional file 2: Supplemental Fig. 2.** F_ST_ for one species pair versus F_ST_ for another species pair.

**Additional file 3: Supplemental Table 1.** Number of F_ST_ windows from the top 1% distributed across different chromosome types for each species pair.

**Additional file 4: Supplemental Table 2.** List of genes associated with top 1% F_ST_ windows determined using genome annotation of Anna’s Hummingbird from Rhie et al. 2020 [74].

**Additional file 5: Supplemental Table 3.** Sex and location for each sample.

**Additional file 6.** Supplementary Methods: Modified Nextera whole-genome library prep protocol for low volumes.

## Data Availability

Raw sequence data is available at NCBI SRA (https://www.ncbi.nlm.nih.gov/sra) under Accession Number PRJNA640148.
