# Supplementary figures and images for: Genomic differentiation across the speciation continuum in three hummingbird species pairs
Source: BMC Evol Biol. 2020 Sep 3;20:113. doi: 10.1186/s12862-020-01674-9 (PMC7469328; doi:10.1186/s12862-020-01674-9)

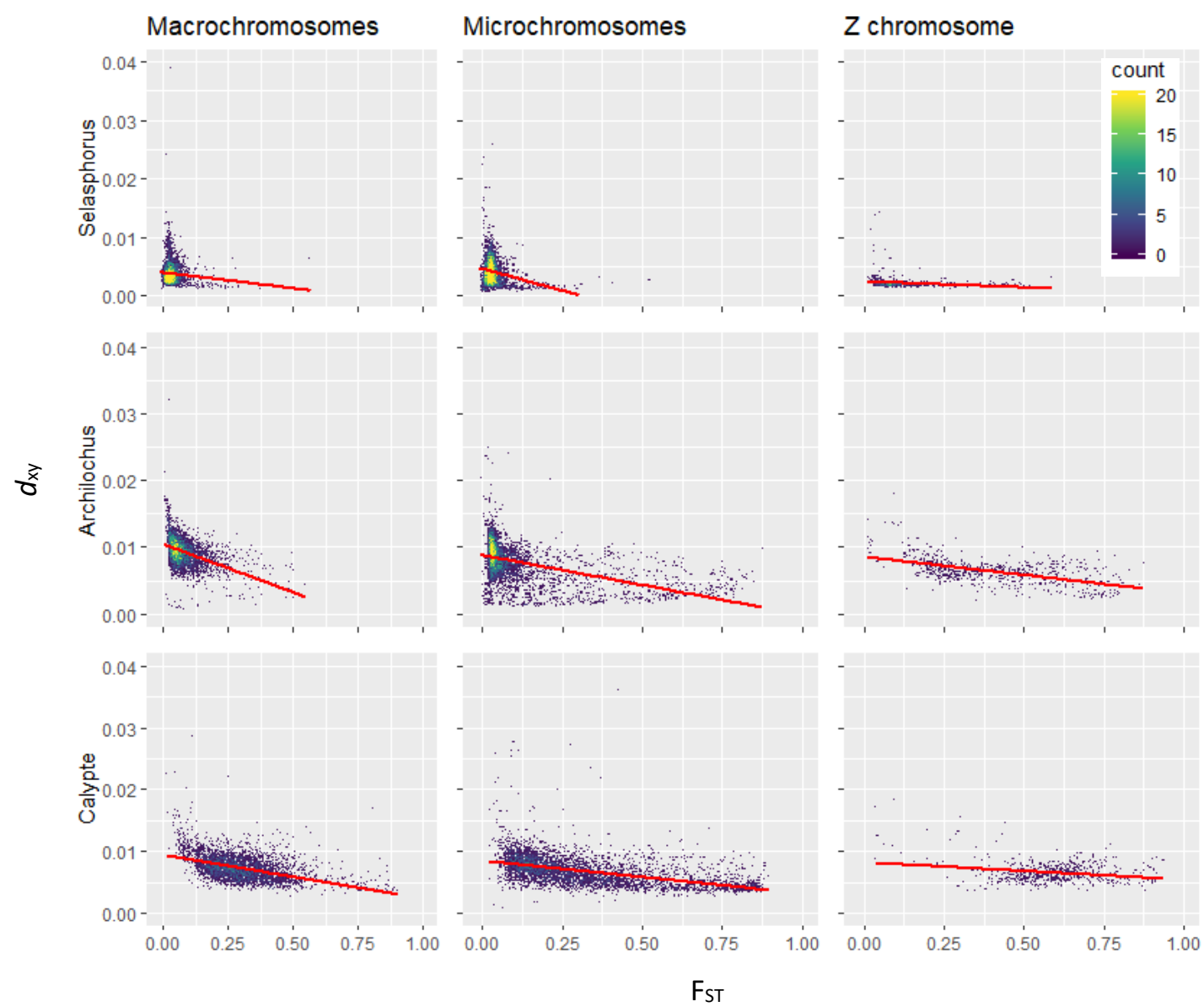

Supplement: Supplementary file 1 — Additional file 1: Supplemental Fig. 1. dxy vs FST. [file 12862_2020_1674_MOESM1_ESM.pdf]

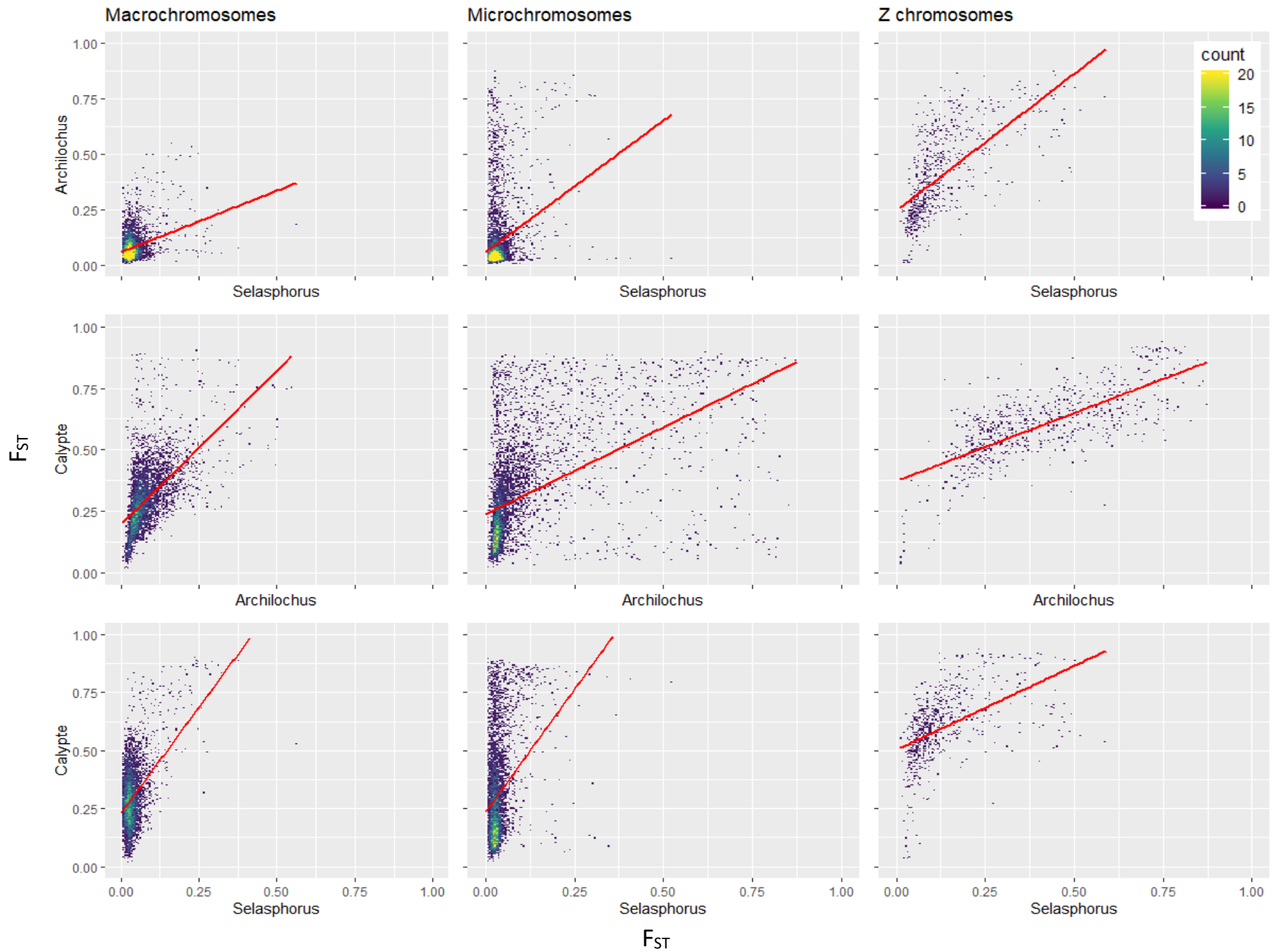

Supplement: Supplementary file 2 — Additional file 2: Supplemental Fig. 2. FST for one species pair versus FST for another species pair. [file 12862_2020_1674_MOESM2_ESM.pdf]
