# Supplementary material for: Genomic differentiation across the speciation continuum in three hummingbird species pairs
Source: BMC Evol Biol. 2020 Sep 3;20:113. doi: 10.1186/s12862-020-01674-9 (PMC7469328; doi:10.1186/s12862-020-01674-9)
Supplement: Supplementary file 3 — Additional file 3: Supplemental Table 1. Number of FST windows from the top 1% distributed across different chromosome types for each species pair. [file 12862_2020_1674_MOESM3_ESM.pdf]

Supplemental Table 1 Top 1% of F_{ST} windows for each species pair and chromosome type.

Species pair	Chromosome type	Total # windows	# windows in top 1%
Selasphorus	Z	720	74
	macro	4635	15
	micro	4850	13
Archilochus	Z	719	49
	macro	4635	0
	micro	4849	53
Calypte	Z	626	17
	macro	4151	16
	micro	3274	47
