# Supplementary material for: Genomic differentiation across the speciation continuum in three hummingbird species pairs
Source: BMC Evol Biol. 2020 Sep 3;20:113. doi: 10.1186/s12862-020-01674-9 (PMC7469328; doi:10.1186/s12862-020-01674-9)
Supplement: Supplementary file 5 — Additional file 5: Supplemental Table 3. Sex and location for each sample. [file 12862_2020_1674_MOESM5_ESM.pdf]

Supplemental Table 3 Sex and location for each sample.

Species	ID	Sex	State	County
A. alexandri	TJF41	F	TX	Brewster
A. alexandri	CJC298	F	TX	Brewster
A. alexandri	CJC297	M	TX	Brewster
A. alexandri	CJC299	M	TX	Brewster
A. alexandri	CJC250	M	TX	Brewster
A. alexandri	CJC249	M	TX	Brewster
A. alexandri	TJF40	M	TX	Brewster
A. alexandri	CJC254	F	TX	Brewster
A. alexandri	CJC300	M	TX	Brewster
A. colubris	TJF169	M	CT	New Haven
A. colubris	VT29	M	CT	New Haven
A. colubris	TJF122	F	CT	Middlesex
A. colubris	CJC310	M	CT	Litchfield
A. colubris	CJC335	F	CT	New Haven
A. colubris	CJC258	M	CT	Fairfield
A. colubris	CJC260	M	CT	New Haven
A. colubris	CJC333	M	CT	Fairfield
A. colubris	CJC334	F	CT	New Haven
S. sasin	CAS96922	M	CA	San Francisco
S. sasin	CAS96923	M	CA	San Francisco
S. sasin	CAS96992	M	CA	San Francisco
S. sasin	CAS97003	M	CA	San Francisco
S. sasin	CAS97088	M	CA	San Francisco
S. sasin	CAS97717	M	CA	San Francisco
S. sasin	CAS97720	M	CA	San Francisco
S. sasin	CAS97760	F	CA	San Francisco
S. sasin	CAS97778	M	CA	San Francisco
S. rufus	CJC371	M	OR	Clatsop
S. rufus	CJC378	M	OR	Clatsop
S. rufus	CJC380	M	OR	Clatsop
S. rufus	CJC382	M	OR	Clatsop
S. rufus	CJC383	M	OR	Clatsop
S. rufus	CJC386	M	OR	Clatsop
S. rufus	CJC387	M	OR	Clatsop
C. anna	CJC001	M	CA	Riverside
C. anna	CJC409	M	CA	Riverside
C. anna	E06964	F	CA	Riverside
C. anna	E06704	F	CA	Riverside
C. anna	E06903	F	CA	Riverside
C. anna	E06904	F	CA	Riverside
C. anna	E06714	M	CA	Riverside
C. anna	E06905	F	CA	Riverside
C. anna	E06906	F	CA	Riverside
C. anna	E06907	M	CA	Riverside
C. anna	E06908	M	CA	Riverside
C. costae	CJC412	F	CA	Riverside
C. costae	CJC418	F	CA	Riverside
C. costae	CC021	M	CA	Riverside
C. costae	CC025	M	CA	Riverside
C. costae	K53206	M	CA	Riverside
C. costae	E06713	F	CA	Riverside
C. costae	E06715	F	CA	Riverside
C. costae	E06716	F	CA	Riverside
C. costae	E06717	M	CA	Riverside
C. costae	E06910	F	CA	Riverside
C. costae	E06911	M	CA	Riverside
C. costae	E06924	F	CA	Riverside
C. costae	E06925	F	CA	Riverside
C. costae	E06926	M	CA	Riverside
C. costae	E06927	M	CA	Riverside
C. costae	CC036	M	CA	Riverside
C. costae	CC045	M	CA	Riverside
