# Supplementary material for: Genomic differentiation across the speciation continuum in three hummingbird species pairs
Source: BMC Evol Biol. 2020 Sep 3;20:113. doi: 10.1186/s12862-020-01674-9 (PMC7469328; doi:10.1186/s12862-020-01674-9)
Supplement: Supplementary file 6 — Additional file 6. Supplementary Methods: Modified Nextera whole-genome library prep protocol for low volumes. [file 12862_2020_1674_MOESM6_ESM.pdf]

Brelsford/Purcell Lab Whole-genome library prep

Draft 3, 5/7/2020

Based on Baym et al. 2015, PLOS ONE.

Modifications to Baym protocol:

1. Module 1: Use 4 ng/ul DNA concentration instead of 0.5
2. Module 2: Tagmentation in 5 ul volume instead of 2.5
3. Module 3: Use NEB Q5 polymerase instead of KAPA master mix
4. Module 3: Increase PCR extension time from 0:30 to 1:30
5. Module 4: use 0.6 bead:sample ratio instead of 1:1.

Detailed Protocol

This protocol is for the preparation of 96 samples (8 rows x 12 columns) but can be modified for either higher or lower throughput.

General tips

- We advise cleaning pipettes and your station with DNA-Away (Thermo Scientific 7010) to reduce contamination from the environment and previous samples.
- Many steps call for centrifugation of 96-well plates. These steps are necessary for consistency across wells when to transferring small volumes of liquid and should not be omitted.
- The potential for cross-contamination of samples and primers, in particular, is high. We recommend the use of filter tips, exclusively aspirating samples or primers with fresh tips, and avoiding “blowing out” the pipette.

Materials and equipment used throughout the protocol

- Sterile DNase-free water
- Filter tips
- Manual multi-channel pipettes. We found less consistent results when mixing with electronic multi-channel pipettes
- DNase-free microfuge tubes, PCR strips, PCR tubes, and PCR 96-well plates
- Centrifuge capable of spinning 96-well plates
- Thermocycler
- (optional) Electronic multi-dispense, multi-channel pipettes
- (optional) 96-well pipette (Liquidator or equivalent)
- (optional) PCR cooler (e.g. Eppendorf 3881)
- (optional) Rubber roller for sealing plates
- (optional) Liquid-handling robot

Module 1. DNA standardization. *Qubit for testing DNA concentrations, when only running a few samples. Target concentration: 4 ng/ul*

Goal.

Obtain at least 5µl of each sample at concentration 4ng/µl (or another chosen concentration)

Procedure: measure DNA conc. (only for many samples of unknown concentration)

1. Follow protocol for Qubit fluorometer.

Procedure: Dilute samples to 4 ng/uL

2. Based on measured DNA concentration of each sample, calculate the volume of water needed to dilute each sample to 4.0 ng/µl.

Use equation: *(C1*V1)/C2 = V2* to calculate the V2, total volume of the final DNA sample at 4 ng/µl. C1 is the measured concentration, C2 is the target concentration (4 ng/µl), V1 is the initial volume (the amount of original DNA added to water for dilution).

Find amount of water to add to each sample using V2 – V1.

3. Dispense these volumes of water into a fresh 96-well plate.
    a. Recommended: Liquid handling robot
    b. If doing manually, a tablet and Pippette-Guide-96 may be helpful (https://github.com/tamilieberman/Pipette-Guide-96).
4. Add **5µl** of gDNA to each well of the new plate.
5. Seal the plate tightly, vortex, and centrifuge (200rcf for 30s).
6. (Optional) Standardized gDNA libraries can be stored overnight at -20°C.

Module 2. Tagmentation

Goal.

Mix 2.5µl of TD buffer, 0.5µl TDE1, and 2µl of gDNA in each well. Final total volume per well is 5µl. Carry out tagmentation reaction in a thermocycler.

Materials and equipment

- Standardized gDNA from Module 1
- Nextera TD buffer and TDE1 enzyme (from Illumina kits FC-121-1030 or FC-121-1031)
- 96-well PCR plate (e.g. Bio-Rad MLP-9601)
- Microseal 'B' (Bio-Rad MSB-1001)

Procedure.

Note: All reagents should be kept on ice. The 96-well plate containing samples should also be kept on ice while assembling the mix, and all steps should be done quickly. Small volume reactions can be difficult to work with; do not skip centrifugation.

1. Preheat thermocycler to 55°C. If starting from frozen gDNA, thaw, vortex and spin down gDNA. Thaw TD buffer and TDE1 on ice.
2. Invert TD buffer and TDE1 gently to mix, spin down, and replace on ice.
3. Make the tagmentation master mix (TMM) by mixing **312µl** buffer and **62.4µl** enzyme in a PCR tube. (*For 8 samples, use **26µl** buffer and **5.2µl** enzyme. Other than 8 samples, use **3.25µl** buffer and **0.65µl** enzyme per sample.)* Mix thoroughly by gently pipetting up and down 10 times.
    a. Excess volumes enable rapid and even distribution of tagmentation enzyme and buffer. It is essential that all samples receive the same amount of enzyme.
4. Distribute TMM into 8-tube PCR strip with **45µl** per tube. Cap and spin down to remove bubbles. (*Draft 1 note: skip this step for testing 8 samples*)
5. Distribute **3µl** of TMM per well into all wells of a fresh PCR plate using a multi-channel pipette.
    a. Dispense into the bottom of the well and ensure the full amount is dispensed each time.
6. Transfer **2µl** of gDNA into each corresponding well using a manual multi-channel pipette. Mix by gently pipetting up and down 10 times.
7. Cover plate with Microseal 'B' and spin down (280rcf for 30s).
8. Incubate in thermocycler for **10min** at **55°C**.
    a. Incubation time between 5 and 20 minutes does not affect the results.
9. Place plate on ice. Allow plate to cool before proceeding to Module 3.

Module 3. PCR-mediated adapter addition and library amplification

Goal.

Mix 22µl of PCR master mix, 8.8µl of index1, 8.8µl of index2, and 5µl of tagmented DNA in each well. Final total volume per well is 45µl.

Materials and equipment

- Tagmented DNA from Module 2
- PCR reagents: Q5 hot-start polymerase, Q5 buffer, high GC enhancer, dNTP 25mM
- Rxxx/Sxxx primers at concentration of 5µM, arrayed in PCR strip (8), S1 Table
- Cxxx/Nxxx primers at concentration of 5µM, arrayed in PCR strips (12), S1 Table
- Microseal 'A' (Bio-Rad MSA-5001) or Microseal 'B' (Bio-Rad MSB-1001)

Procedure. *(Written for a small batch of samples. Before scaling up, incorporate the high-throughput layout of primers from Baym et al.)*

1. Make PCR master mix as follows, keeping the master mix and all reagents on ice. Recalculate third column for your specific number of samples:

Reagent	Number of samples<br>1x	8x * 1.1
Water	3.18	30.53
Q5 buffer	9	86.4
High GC enhancer	9	86.4
dNTP **(25mM)**	0.36	3.46
Q5 hot-start polymerase	0.46	4.42

2. Add the following to each well of the tagmentation plate which contains 5µl tagmented DNA per well: 22 µl master mix, 8.8µl of index1, 8.8µl of index2. Mix and spin down.
3. Run the following program:
    1. 72°C for 3 min
    2. 98°C for 5 min
    3. 98°C for 10 sec
    4. 63°C for 30 sec
    5. 72°C for 1 min
    6. Repeat steps (3)-(5) 13 times for total of 13 cycles
    7. 72°C for 5 min
    8. Hold at 12°C
4. Transfer the PCR products to post-PCR lab. Optional: if using Microseal 'B', the plate can be left at 4°C overnight.

Module 4. PCR clean-up and size selection

Materials and equipment.

- Tagmented and indexed DNA
- 100% ethanol
- Resuspension buffer (10mM Tris-Cl [pH 8.0] + 1mM EDTA [pH 8.0] + 0.05% Tween-20)
- Deep-well 96-well plate for bead purification
- Magnetic beads for DNA purification (2% v./v. Sera-Mag SpeedBeads, 18% w./v. PEG-8000, 1M NaCl, 10mM Tris HCl, 1mM EDTA, 0.05% Tween 20; e.g. according to http://ethanomics.files.wordpress.com/2012/08/serapure_v2-2.pdf)
- 96-well plate magnetic stand (e.g., Life Technologies, Cat. #123-31D).

Procedure.

Note: It is best to thaw beads at the beginning of Module 1, as it takes time for them to reach room temperature. While at this stage cross-contamination is a much smaller issue, we still recommend fresh tips for each well.

Draft 1 note: batch quantities to place in reservoirs below are for an entire 96-plate; reservoirs are probably not necessary for testing batches of 8 samples

1. Centrifuge the PCR plate at 200rcf for 30 seconds.
2. To resuspend beads, alternate between vortexing and inverting beads for a total of at least 60 sec.
3. *(Draft 1 Note: only for an entire 96-plate!)* Transfer at least **5ml** of beads into a reagent reservoir.
4. Using a multi-channel pipette, transfer **27µl** of beads into each well of the PCR plate and pipette up and down several times to mix.
    a. Pipette into the bottom of wells and ensure that the beads are completely dispensed.
    b. Some prefer to purify in a separate deep-well 96-well plate, for easier aspiration. In this case, we recommend first adding 15µl of beads to each well of the deep-well plate, then transferring 15µl of sample into each well and mixing.
5. Incubate at room temperature for **5 min**. DNA is now on the beads.
6. *(Draft 1 Note: only for an entire 96-plate!)* Prepare a fresh batch of 80% ethanol by mixing **10mL** of sterile water and **40mL** of 100% ethanol in a sterile reservoir.
7. Place the plate on the magnetic stand and incubate for **1 min** to separate beads from solution. The solution should become clear.

8. While the plate is on the magnetic stand, aspirate clear solution from the plate and discard. Do not disturb the beads. If beads are accidentally aspirated, resuspend them, wait 1 min, and aspirate again.
9. While the plate is on the magnetic stand, dispense **200µl** of 80% ethanol into each well. Incubate for **1 min** at room temperature.
10. Aspirate ethanol and discard. Do not disturb the beads. If beads are accidentally aspirated, resuspend them, wait 1 min, and aspirate again.
11. Repeat steps 9–10 for a total of 2 washes.
12. Remove any visibly remaining ethanol droplets with smaller pipette tips.
13. Let the plate air dry for **20 min** for residual ethanol to evaporate.
14. *(Draft 1 Note: only for an entire 96-plate!)* Transfer at least **3.5ml** of resuspension buffer to a new reservoir.
15. Take the plate off the magnetic stand. Add **30µl** of resuspension buffer to each well of the plate using a multichannel pipette. Resuspend the beads by mixing 10–15 times.
16. Incubate for **5 min** at room temperature. DNA is now in the solution.
17. Place the plate back onto the magnetic stand and incubate for about **1 min** to separate beads from solution. The solution should become clear.
18. While the plate is on the magnetic stand, aspirate clear solution from the plate and transfer to a fresh 96-well plate. Do not disturb the beads. If beads are accidentally pipetted, resuspend them, wait for the solution to become clear, and repeat.
19. Seal plate and spin down (200rcf for 30s).
20. (Optional) DNA libraries can be stored at -20°C before proceeding to Module 5.

Module 5. Library QC and Pooling

Materials and equipment. *Use Qubit (steps 1-2) for quantification when working with small number of samples.*

- Purified Nextera libraries from Module 4
- High Sensitivity DNA kit for BioAnalyzer (Agilent 5067–4626)
- TE buffer
- 50mL reagent reservoirs
- Qubit fluorometer

Procedure.

1. Perform steps 1–7 of Module 1 to quantify DNA concentration across all samples.
    a. You may use less DNA and ladder (5 or 8 µl) to conserve sample.

2. Calculate the concentration of each sample. We typically discard samples with low concentrations (< 0.5ng/µl), which would not have enough coverage and would dilute the final concentration of pooled samples.
3. Run 7µl of each sample on a 1.5% agarose gel for 45min.
4. Pool acceptable samples in equimolar concentrations.
5. If sequencing in-house, accurate quantification is crucial to achieve optimal cluster density. We recommend using qPCR (KAPA KK4824) and running a BioAnalyzer on the final pooled library.
6. The pooled library should be stored at -20°C.
